# What is wrong with sleep in ADHD? An overview of current knowledge about sleep and ADHD and the impact of treatment on ADHD.

**DOI:** 10.1192/j.eurpsy.2026.11523

**Published:** 2026-07-31

**Authors:** J. S. Kooij

**Affiliations:** 1 Psychiatry, Amsterdam UMC/VUMc, Amsterdam; 2 Adult ADHD, PsyQ, the Hague, Netherlands

## Abstract

**Introduction:**

Sleep onset and maintenance difficulty is highly prevalent in adults with ADHD, as it is in the youth, especially delayed sleep phase disorder, insomnia and restless legs syndrome.

**Objectives:**

In this talk, the most frequent sleep disorders will be discussed and their relationship with ADHD as a neurobiological developmental disorder.

**Methods:**

According to the current literature, the most frequent sleep disorder in ADHD is the Delayed Sleep Phase Disorder, with a prevalence up to 80% across the lifespan. Other disorders with increased prevalences are insomnia, restless legs, and sleep apnea.

**Results:**

The high prevalence of the Delayed Sleep Phase Syndrome suggests ADHD may be a 24-hour disorder with symptoms occurring both during the day and during the night. As a result, sleep and ADHD should both be addressed during treatment.

Screening for sleep disorders needs to be a part of routine assessment of ADHD in adults, for instance with the Holland Sleep Disorder Questionnaire, and monitored throughout treatment. Besides late sleep, many patients have more than one sleep disorder, so all sleep disorders need to be addressed in order to achieve a good sleep quality.

Chronotherapy, that is treatment with sleephygiene plus timed low dose melatonin, and bright light therapy in the morning has been found effective for improving not only the delayed onset of sleep, but also mood and ADHD symptoms.

**Conclusions:**

ADHD and sleep disorders often come together and should be treated in concert, as treatment of certain sleep disorders also improves ADHD symptoms.

**References:**

- van Andel E, Bijlenga D, Vogel SWN, Beekman ATF, Kooij JJS. Effects of chronotherapy on circadian rhythm and ADHD symptoms in adults with attention-deficit/hyperactivity disorder and delayed sleep phase syndrome: a randomized clinical trial. *Chronobiol Int.* Feb 2021;38(2):260-269.
- van Andel E, Bijlenga D, Vogel SWN, Beekman ATF, Kooij JJS. Attention-Deficit/Hyperactivity Disorder and Delayed Sleep Phase Syndrome in Adults: A Randomized Clinical Trial on the Effects of Chronotherapy on Sleep. J Biol Rhythms. 2022 Dec;37(6):673-689.
- van der Ham M, Bijlenga D, Böhmer M, Beekman ATF, Kooij S. Sleep Problems in Adults With ADHD: Prevalences and Their Relationship With Psychiatric Comorbidity. J Atten Disord. 2024 Nov;28(13):1642-1652.
- Bijlenga D, Vollebregt MA, Kooij JJS, Arns M. The role of the circadian system in the etiology and pathophysiology of ADHD: time to redefine ADHD? Atten Defic Hyperact Disord. 2019 Mar;11(1):5-19.
- Fargason RE, Fobian AD, Hablitz LM, Paul JR, White BA, Cropsey KL, Gamble KL. Correcting delayed circadian phase with bright light therapy predicts improvement in ADHD symptoms: A pilot study. J Psychiatr Res. 2017 Aug;91:105-110.

**Disclosure of Interest:**

None Declared

